# Reliability of the Frontal Plane Knee Alignment Measurement Based on a Remote Protocol

**DOI:** 10.5195/ijt.2022.6506

**Published:** 2022-12-13

**Authors:** Karina Rodrigues Mauro, Laura de Menezes Cantusio, Karina Guimarães de Brito Menezes, Karine Jacon Sarro

**Affiliations:** School of Physical Education, University of Campinas, São Paulo, Brazil

**Keywords:** Dynamic knee valgus, Functional test, Kinematics, Movement evaluation

## Abstract

**Introduction:**

The analysis of movement quality is important for better exercise prescription. This study tested the reproducibility of a protocol for remote assessment of dynamic knee alignment using images taken by patients.

**Methods:**

Thirteen women filmed themselves performing single-leg squats on two days at a 15-day interval. Three raters measured the knee frontal plane projection angle using the resultant images.

**Results:**

Two participants (15.4%) were excluded for not performing the protocol correctly. The intraclass correlation coefficient was between 0.880 and 0.999 for the intra-rater comparison, and between 0.817 and 0.987 for the inter-rater comparison.

**Discussion:**

The success of the protocol in 84.6% of participants and the excellent reproducibility suggest that the methodology of analyzing patient-captured cell phone images might be a plausible alternative for remote evaluation of dynamic knee alignment.

In the past years, an increasing number of healthcare systems are adopting telemedicine and telerehabilitation as alternative methods of healthcare delivery to patients in remote areas or due to the need for social distancing, as during the worldwide COVID-19 pandemic in 2020. Technological advances have considerably improved clinicians' capability to deliver rehabilitation remotely (i.e., via the internet) and several studies have shown its effectiveness (Jiang et al., 2018; Kairy et al., 2009; Mani et al., 2017; Palacín-Marín et al., 2013). However, physical examination is challenging in a telerehabilitation setting—especially the evaluation of movement.

People with altered movement patterns may have reduced functional performance and might be at increased risk for musculoskeletal injury. More specifically, inadequate muscular control of the hip, pelvis, and trunk in a closed kinetic chain affects the kinematics and kinetics of the tibiofemoral and patellofemoral joints, increasing the medial motion of the knee. This may be a possible risk factor for knee injuries, such as patellofemoral pain, rupture of the anterior cruciate ligament, and iliotibial band syndrome (Brechter et al., 2003; Hewett et al., 2005; McLean et al., 2005; Powers, 2003). Although these injuries can affect men, women, and individuals of all ages, they are more common in young people and in the female population (Barrios et al., 2016; Crowell, Nokes & Cosby, 2021). Therefore, the use of functional tests to evaluate basic motion patterns and movement control is essential to rehabilitation and exercise program planning.

Specific assessment of individuals who demonstrate excessive medial knee motion during functional tasks may allow the identification and modification of altered movement patterns, based on prescription of specific exercises to increase muscle force and control. Although observational movement screening tests offer a cost-effective, time-efficient method of assessing gross movement patterns, the results are subjective, and this subjectivity could be even higher when not done in person.

Therefore, the use of quantitative biomechanical measures promises greater accuracy in a telerehabilitation setting.

Although the gold standard for biomechanical evaluation of movement is the three-dimensional (3-D) kinematic analysis, two-dimensional (2-D) video analysis provides healthcare professionals with a useful tool that is portable, has a low cost, and is readily available. Gwynne and Curran (2014) found that 2-D measurements have good consistency and can provide valid measures of lower limb alignment when compared to 3-D methods.

A 2-D measure widely used for the quantitative analysis of knee alignment during functional tasks is the frontal plane projection angle (FPPA), which is the angle formed between the thigh and the shank in the frontal plane, generally measured during knee flexion in a closed kinetic chain task (Munro et al., 2012). This appears to be a consistent biomechanical parameter, that could be effectively utilized to evaluate knee injury risk during the single leg squat (SLS). The SLS is a movement regularly used in clinical practice as it approximates knee motion in common activities and is often pain provoking (Werner et al., 2019). The measurement of knee FPPA historically involves the positioning of surface markers against anatomical landmarks to identify their positions through the movement (Gwynne & Curran, 2014; Munro et al., 2012; Werner et al., 2019). In addition, studies that verified the reproducibility of this method were carried out in controlled environments, such as motion analysis laboratories (Gwynne & Curran, 2014; Munro et al., 2012; Werner et al., 2019). This equipment and methodology would be impossible in a telerehabilitation scenario.

A recent systematic review found that there is little evidence regarding the application of musculoskeletal assessment methods remotely, indicating the need to adapt existing tests (Murray et al., 2021). The 2D assessment of knee alignment from the knee frontal plane projection angle may be a promising method for remote use, if it can be performed without the use of markers, and using images directly produced by the patient. This study aimed to verify the reproducibility of a protocol to evaluate knee alignment remotely using measurement of the knee FPPA without using surface markers and using patient-provided cell phone video images.

## Methods

The study enrolled 13 women (24±3.5 years of age) who were physically active and without lower limb injuries. After two subjects were excluded for inability to follow the study protocol, a total of 22 knees were analyzed. The study was approved by the Human Research Ethics Committee of the university where the study was conducted (approval number 3.983.878; CAAE 46484121.9.0000.5404). Informed signed consent was obtained from all participants prior to data collection.

The participants were instructed to record themselves with their own cell phones while executing single leg squats and email the videos to the researchers. Participants first watched a video that detailed the specific required position of the cell phone as well as the specifics of the single leg squat. The cell phone was positioned at a height of approximately 60 centimeters and at a distance of 1.5 meters from a strip of adhesive tape marking the foot position where the squat would be performed, supported in such a way that it was not tilted. They were instructed to use books, boxes, or other objects to achieve the correct position for the cell phone. They then stood on one leg facing the cell phone, with the foot of that leg positioned immediately behind the adhesive tape and with the hands on the waist. They then squatted/flexed the hip and knee with an erect trunk until the knee was directly over the adhesive tape (approximately 45 degrees of knee flexion), and then returned to the initial position. The squat was to be performed over a 5 second interval: the movement initiates at the start of the interval, the lowest point of the squat is reached at the third second, and the start position is reached at the fifth second (Munro et al., 2012). The squat was repeated three times with each leg on an alternating basis. The participants were encouraged to practice the squat three times before recording the movement.

**Figure 1:**
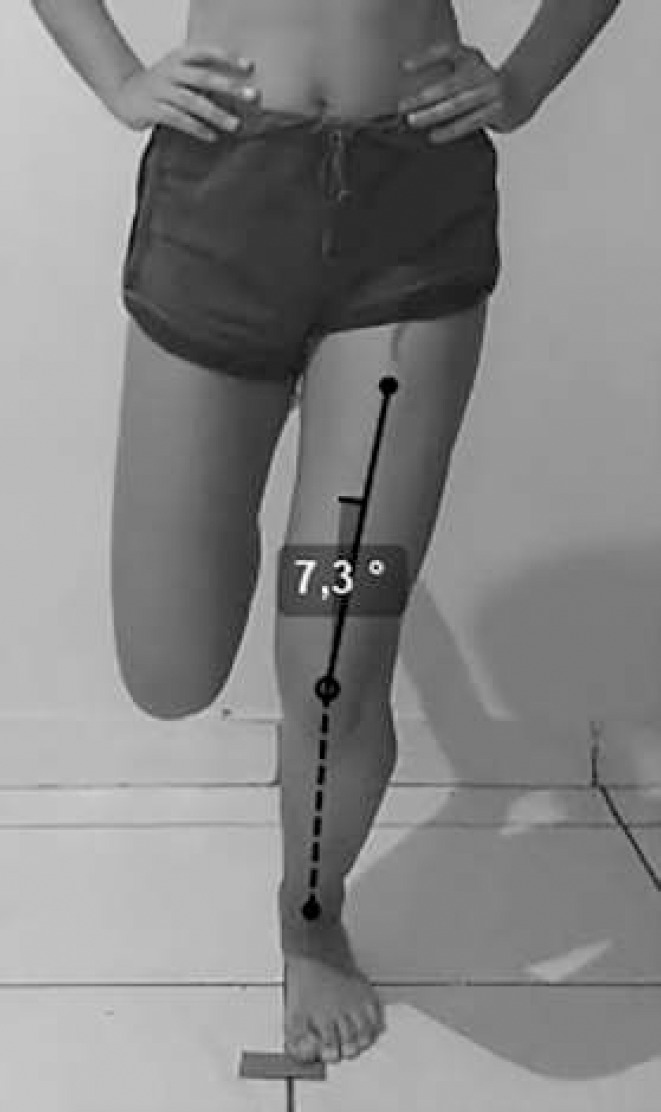
Measurement of the Knee Frontal Plane Projection Angle

The resultant video files were analyzed by three raters. Two raters were physical therapists, and one was a physical education professional. The frontal plane projection angle of the knee (FPPA) was measured using the software Kinovea® 0.9.4 (open source) at the point of maximal knee flexion, which was estimated visually by the rater. The FPPA, which characterizes knee alignment, is defined by the angle between the anterior superior iliac spine, the midpoint between the femoral condyles (center of the knee) and the midpoint between the malleoli (center of the ankle) (Munro et al., 2012). Because it is challenging to locate the anterior superior iliac spine via video without prepositioned anatomical marker devices, the center of the thigh in its middle third was used as a reference instead (Figure 1).

**Figure 2:**
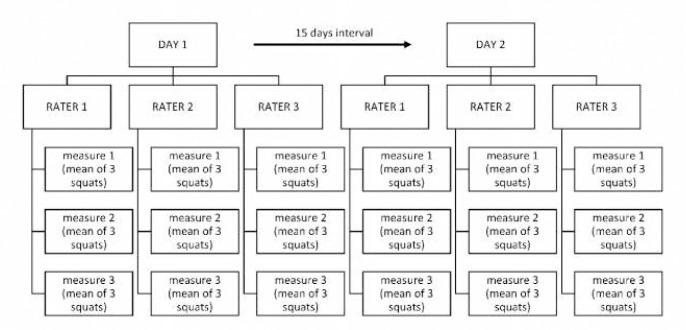
Diagram Illustrating the Measurement Procedure

To evaluate the reproducibility of the FPPA measured without surface markers, the three raters measured each squat repetition of each knee of each participant, three times. The same measurement procedure was repeated 15 days after the first day of data collection. All raters were blinded to other raters' measurements, as well as their own previous measurements. To minimize intrasubject variability, the mean of the three repetitions of the same side on a given participant was analyzed (Figure 2).

The intra-rater reproducibility was evaluated by the intraclass correlation coefficient, based on a two-way mixed effect, absolute agreement model (ICC(3,1)). The interrater reproducibility was evaluated by the intraclass correlation coefficient based on a two-way random effects, absolute agreement model (ICC(2,1)). The reproducibility would be classified as poor if the ICC were less than 0.5, moderate if the ICC were between 0.5 and 0.75, good if between 0.75 and 0.9 and excellent if greater than 0.9.

## Results

Two participants did not execute the protocol correctly and were excluded: one performed only two repetitions, and one did not alternate the right and left sides. Therefore, 22 knees were analyzed.

**Figure 3.**
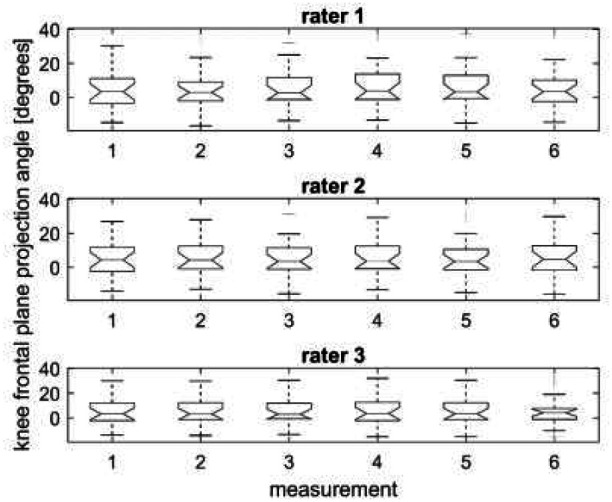
Knee Frontal Plane Projection Angle Measured by Three Raters on Two Different Days

Note. Measurements 1 to 3 – day 1; measurements 4 to 6 – day 2

The results of the knee FPPA measured by the three raters on two different days are shown in Table 1 (mean and standard deviation) and in Figure 3 (boxplot). The results are very similar, with a large standard deviation, showing the high variability of the FPPA among the participants (Table 1).

**Table 1.** Mean and Standard Deviation of the Knee Frontal Plane Projection Angle of 22 Knees Measured by Three Raters, Three Times, on Two Different Days

	Day 1			Day 2		
	Measure 1	Measure 2	Measure 3	Measure 1	Measure 2	Measure 3
**Rater 1**	3.89 (±11.12)	4.40 (±11.79)	4.74 (±11.81)	5.49 (±11.92)	6.07 (±11.71)	4.54 (±11.94)
**Rater 2**	4.04 (±10.29)	4.50 (±10.00)	4.15 (±11.05)	4.46 (±10.70)	4.15 (±11.07)	4.40 (±10.89)
**Rater 3**	4.70 (±10.85)	5.26 (±10.10)	5.14 (±9.91)	5.08 (±11.17)	5.13 (±10.03)	4.52 (±9.77)

In Figure 3, the central horizontal line through the box represents the median, and the notches surrounding the medians represent the 95% confidence interval, providing a measure of the rough significance of differences between the values (McGill et al., 1978). The horizontal dashes that limit the box below and above represent, respectively, the lower and upper quartiles, while the horizontal dashes after the dashed vertical lines are the minimum and maximum values. No difference was found between the measurements since the notches about the medians overlap.

Considering the interrater analysis (Table 2), good-to-excellent reproducibility was found for both days (ICCs between 0.817 and 0.966). With respect to intrarater analysis (Table 3), excellent reproducibility was found for both days across all three raters, with ICCs higher than 0.960.

**Table 2.** Interrater Reproducibility – Intraclass Correlation Coefficient (Confidence Interval)

	Day 1	Day 2
**Measure 1**	0,969 (0,938–0,986)	0,970 (0,942–0,987)
**Measure 2**	0,931 (0,868–0,969)	0,903 (0,817–0,955)
**Measure 3**	0,938 (0,880–0,972)	0,927 (0,858–0,966)

**Table 3.** Interrater Reproducibility – Intraclass Correlation Coefficient (Confidence Interval)

	rater 1	rater 2	rater 3
Day 1	0,981 (0,962–0,992)	0,986 (0,972–0,994)	0,981 (0,962–0,991)
Day 2	0,970 (0,938–0,986)	0,995 (0,990–0,998)	0,966 (0,933–0,985)
Day 1 vs day 2 (measure 1)	0,976 (0,880–0,992)	0,990 (0,976–0,996)	0,997 (0,992–0,999)
Day 1 vs day 2 (measure 2)	0,960 (0,881–0,985)	0,985 (0,964–0,994)	0,994 (0,985–0,997)
Day 1 vs day 2 (measure 3)	0,972 (0,934–0,988)	0,994 (0,986–0,998)	0,965 (0,919–0,985)

## Discussion

This study aimed to verify the reproducibility of a protocol to evaluate knee alignment remotely by measuring of the knee FPPA without markers, using video images generated by the patient using a cell phone. The results show excellent reproducibility, both with respect to the measurements of the same rater as well as the measurements of different raters.

Knee alignment is frequently assessed visually in clinical practice. However, considering in-person evaluations, the reliability of this qualitative assessment varies from poor to moderate (Ressman et al., 2021; Simon et al., 2018). Furthermore, a strong correlation was found between FPPA and hip adduction during 3D analysis, reinforcing the advantages of the 2D evaluation (Simon et al., 2018).

The reliability of the knee 2D FPPA has already been evaluated in a controlled environment. For protocols that used surface markers, good to excellent reliability was found (Gwynne & Curran, 2014; Munro et al., 2012). Tate et al. (2015) evaluated a protocol without surface markers with the video captured by novice and expert raters, and found excellent reliability, with ICCs between 0.91 and 0.96. This agrees with our results (ICCs between 0.82 and 0.99) obtained in an uncontrolled environment with video captured by the subjects themselves.

The reliability found in the present study also matches the reliability of internet-based tools, such as internet-based goniometers. Charlebois et al. (2000) and Russell et al. (2003) investigated the intra- and interrater reliability of a videoconference-based goniometer to measure knee angle, generating ICCs higher than 0.96. However, videoconference-based protocols are dependent on the quality of the Internet connection, which may be a problem in remote areas. Problems with connectivity as well as audio and video quality have already been identified as key issues (Charlebois et al., 2000; Russell et al., 2003). The protocol presented in the present study solves this problem, since it is not based on real-time evaluations, but rather on patient self-assessment recorded directly to a file in the recording device.

Regarding the knee FPPA values, the mean values found are between 3.9° and 6.1°. These values are in agreement with the results found by Tate et al. (2015), between 3.2° and 5.3°, but are slightly different from the values found in studies using surface markers, such as 11.07° (Munro et al., 2012) and ‒7.8° (Gwynne & Curran, 2014). This difference could be associated with the fact that Gwynne and Curran (2014) used markers attached at the anterior superior iliac spine (ASIS). The more lateral position of the markers on the pelvis, subsequently generated values were inherently higher than ones generated with our protocol. Furthermore, although it is unclear if a larger flexion angle could increase the FPPA, some other protocols utilized a larger knee flexion angle. We asked participants to squat only so far until the knee would cross a small marker in front of the foot, expecting to reach around 45 degrees of knee flexion. The authors cited (Gwynne & Curran, 2014; Munro et al., 2012) specified a minimum of 45° and 60° flexion angle, respectively.

The protocol has some limitations. The ability of the patient to correctly and consistently set-up the cell phone camera and collect the images is crucial, limiting the use of the protocol to people without cognitive impairments and to those familiar with generating video using a cell phone. In addition, considering that the study participants were asymptomatic young volunteers, the results cannot be generalized to other populations, since the range of motion and number of squat repetitions may be a challenge. As in the in-person evaluation, single-leg squats are more indicated to assess athletes and young active people. For persons with disabling pain or reduced functionality conditions, such as elderly individuals, less complex tests are more indicated to guide therapeutic and training choices. Therefore, protocol adaptations need to be further explored in this population.

This study did establish on a pilot basis in a limited population, that cell phone images taken by the patients can be used to evaluate knee frontal plane projection angle. This suggests it is a feasible tool for the remote assessment of knee movement in sports medicine practice.
